# Supplementary material for: Amyloid-β Tetramers and Divalent Cations at the Membrane/Water Interface: Simple Models Support a Functional Role
Source: Int J Mol Sci. 2023 Aug 11;24(16):12698. doi: 10.3390/ijms241612698 (PMC10454299; doi:10.3390/ijms241612698)
Supplement: Supplementary file 1 [file ijms-24-12698-s001.zip › ijms-2510201-supplementary.pdf]

Amyloid-β tetramers and divalent cations at the membrane/water interface: Simple models support a functional role.

Authors: Pawel Krupa [1], Giovanni La Penna [2,3,*] and Mai Suan Li [1,4]

[1] Institute of physics, Polish Academy of Sciences, Warsaw, Poland;
[2] Istituto di Chimica dei Composti Organometallici, Consiglio Nazionale delle Ricerche (CNR), Sesto Fiorentino (Firenze), Italy;
[3] Sezione di Roma Tor Vergata, Istituto nazionale di fisica nucleare, Via della Ricerca Scientifica 1, I-00133 Roma, Italy;
[4] Institute for Computational Science and Technology, Ho Chi Minh City, Vietnam.

Supplementary data

- Figure S1 - Minimal distance between addressed cation and any of the atoms in DMPC measured in cMD along with time.
- Figure S2 - Radial distribution function ($g(r)$) of M-OX pairs, with OX any oxygen atom in DMPC, measured in cMD.
- Figure S3 - Distribution of $\mathrm{SASA_{PM}}$ and $\mathrm{SASA_{P}}$ measured for models **1-3**/DMPC, in the differ-ent cMD trajectories (1-5).
- The frequency of values of R $\geq$0.05 is also reported for each separated trajectory (right) in Fig. 3.

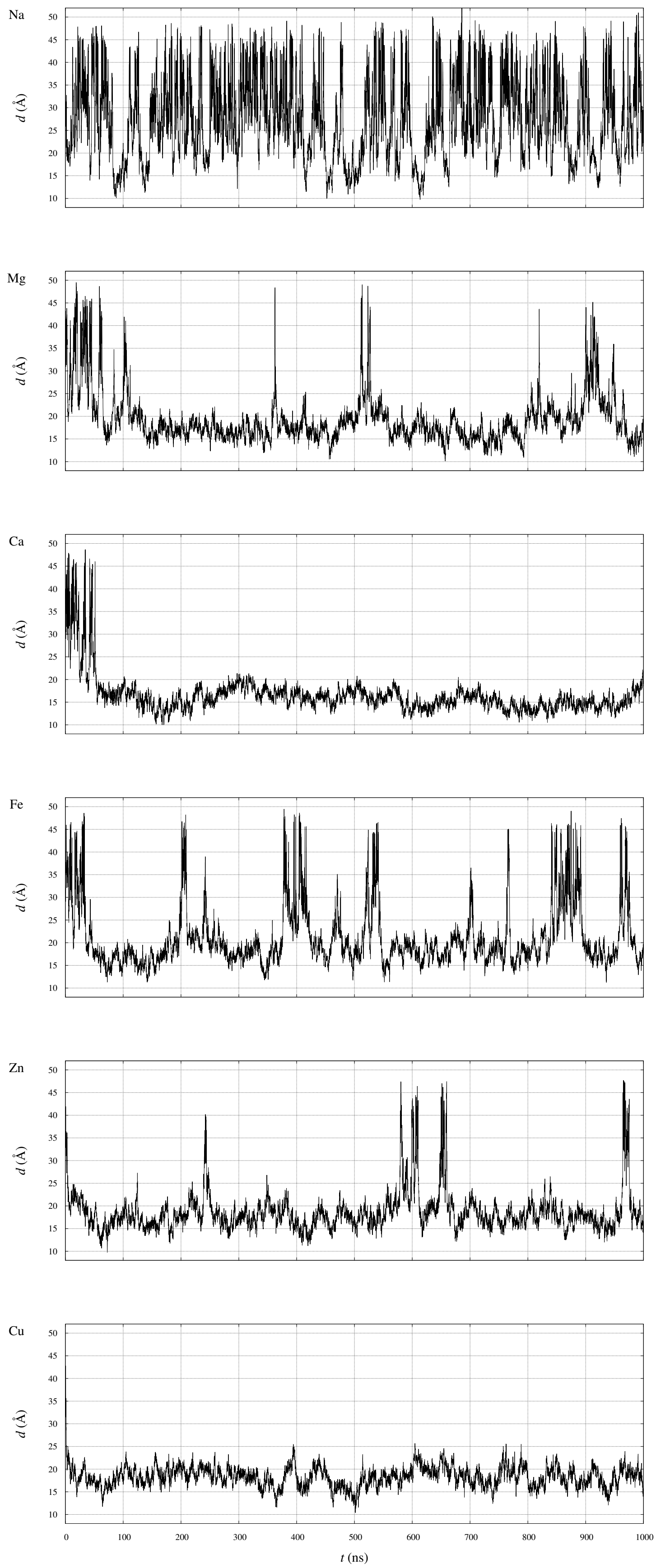

Figure S1: Minimal distance (d) between addressed cation M and any of the atoms in DMPC as mea-sured in cMD simulations along with time t.

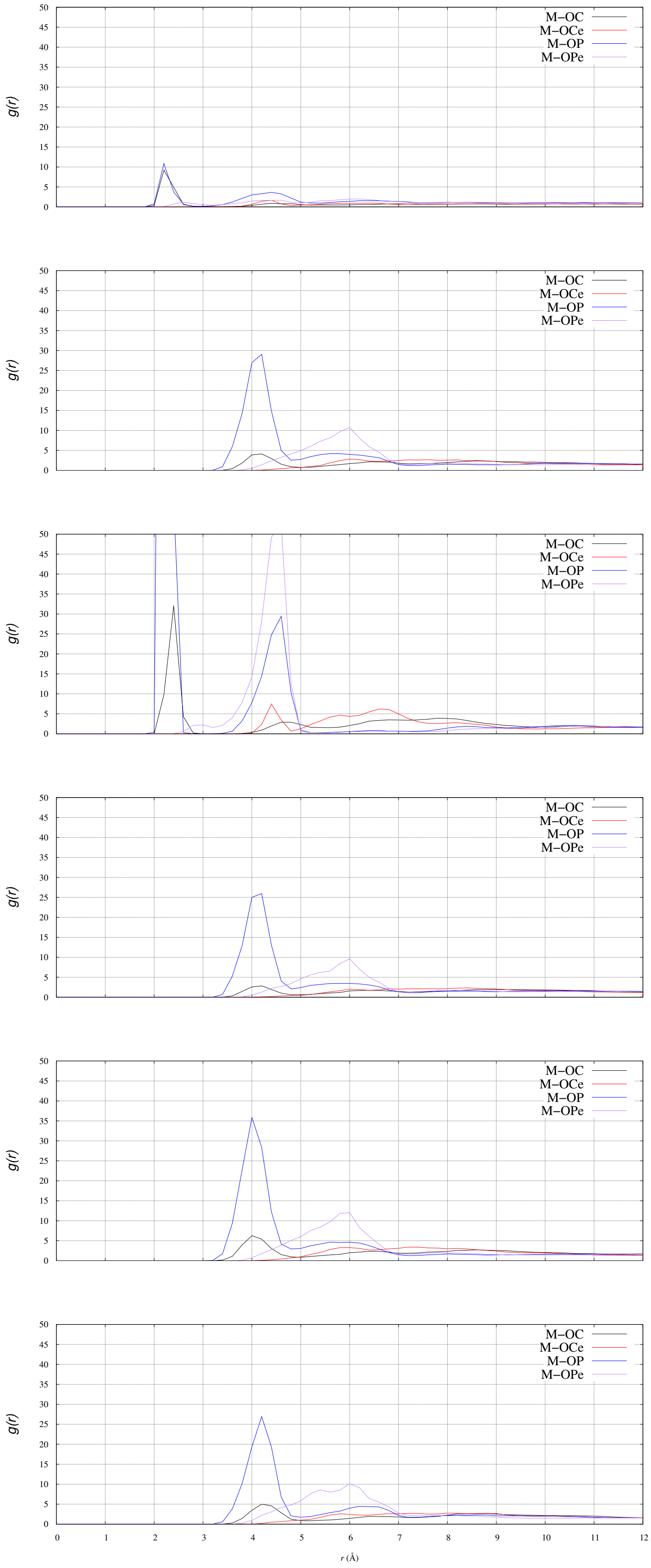

Figure S2: Radial distribution function ($g(r)$) of M-OX pairs, with M the addressed cation and OX the O atoms in DMPC, measured in cMD simulations. M-OC (black); M-OCe (red); M-OP (blue); M-OPe (purple). OC is the carbonyl oxygen; OCe is the ester oxygen; OP is the terminal phosphodiester oxygen; OPe is the bridging phosphodiester oxygen.

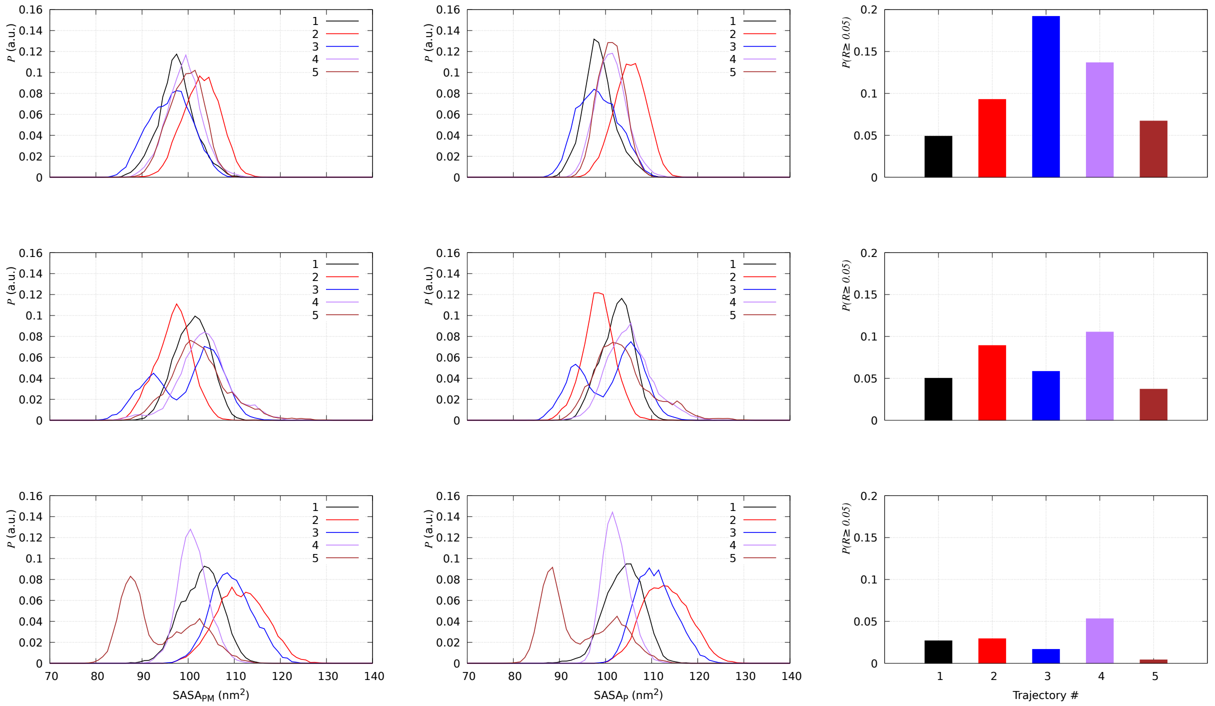

Figure S3: Distribution of SASA$_{\rm PM}$ (left) and SASA$_{\rm P}$ (middle) (see Methods) of A 42 tetramers in the different models exposed to DMPC. Right - Frequency of samples with $R \geq 0.05$ for each trajectory (x-axis). The different curves (1-5) are obtained by the different independent cMD trajectories (see Table 2 in the main text). Top - model **1**/DMPC; middle - **2**/DMPC; bottom - **3**/DMPC.
